# Brief cognitive assessment in a UK population sample – distributional properties and the relationship between the MMSE and an extended mental state examination

**DOI:** 10.1186/1471-2318-5-7

**Published:** 2005-05-04

**Authors:** Felicia A Huppert, Sara T Cabelli, Fiona E Matthews, the MRC Cognitive Function and Ageing Study (MRC CFAS)

**Affiliations:** 1 Department of Psychiatry, University of Cambridge, Cambridge, UK; 2 Department of Public Health & Primary Care, Institute of Public Health, University of Cambridge, Cambridge, UK; 3 MRC Biostatistics Unit, Institute of Public Health, Cambridge, UK

## Abstract

**Background:**

Despite the MMSE's known flaws, it is still used extensively as both a screening instrument for dementia and a population measure of cognitive ability. The aim of this paper is to provide data on the distribution of MMSE scores in a representative sample from the UK population and to compare it with an extended cognitive assessment (EMSE) which covers a wider range of cognitive domains and provides a wider range of difficulty levels.

**Methods:**

The MMSE and the EMSE were administered to over 12,000 participants at the screening stage of the MRC Cognitive Function and Ageing Study (MRC CFAS). MRC CFAS is a multi-centre population-based study in England and Wales with respondents aged 65 years and older.

**Results:**

Normative values on the MMSE and EMSE are presented by age group, sex and level of education. There are very large differences between age groups, with smaller differences seen between the sexes and by level of education. The EMSE extends the scores at the high end of the ability range, but is no better than the MMSE at differentiating between dementia and non-dementia.

**Conclusion:**

Population-derived norms are valuable for comparing an individual's score to the score that would be expected among the general population, given the individual's specific demographic characteristics.

## Background

The Mini-Mental State Examination (MMSE) was developed almost 30 years ago as a screen for dementia among hospital patients[1]. It remains the most widely used short cognitive test in clinical practice, clinical research, and epidemiological studies [2,3] However, its shortcomings are well known [4-6]. Principal amongst them are (a) very limited coverage of memory function, (b) a ceiling effect, resulting in inability to differentiate moderate from high functioning, and (c) absence of information about some aspects of cognitive function required for dementia diagnosis using internationally agreed criteria (DSM-IV [7]; ICD-10 [8]), namely perception and executive function. There is therefore a need for a short screening test that covers the range of cognitive processes required by diagnostic criteria, avoids the ceiling effect, and has an improved coverage of memory.

The Modified Mini-Mental State (3MS) Examination [4] was developed to extend the range of items in the MMSE and avoid ceiling and floor effects. It added eight extra items to the 19 items of the MMSE (date and place of birth, counting backwards from five to one, naming a body part, an easy repetition item, animal naming, a similarities item, and a delayed recall test), as well as a much extended scoring range for the original MMSE items, increasing the total score from 30 to 100. While the additional items extend the coverage of the MMSE by assessing additional aspects of cognitive function, i.e. remote memory (date and place of birth) and executive functioning (animal naming, similarities), the additional items are mainly designed for the low end of the ability range rather than the high end. Moreover, the 3MS still omits the assessment of perceptual ability which is required by diagnostic criteria to establish whether there is evidence of agnosia (DSM-IV [7]; ICD-10[8]). A large population-based study comparing the MMSE and the 3MS in a Canadian sample of people aged 65 years and over reported that the superiority of the 3MS over the MMSE appears more due to its extended scoring system than to its additional questions[9]. The 3MS has an additional drawback: it uses non-standard versions of some of the MMSE items and additional items. Specifically, for the memory task, rather than using three high frequency object nouns such as "apple – table – penny" which became standard in the community version of the MMSE[10], the 3MS uses the words "shirt – brown – honesty", which are more difficult to remember as they do not form a single visual image. Also the MMSE item asking subjects to write a sentence of their own choosing has been replaced in the 3MS by writing a sentence to dictation, which is far easier as it does not require the subject to generate a sentence. With regards to the additional items, the animal naming task gives the subject 30 seconds to name 4-legged animals, in contrast to the standard semantic fluency task which allows one minute to name any animals. Likewise, the 3MS uses a non-standard similarities question, ("In what way are an arm and a leg alike?"). These deviations mean that the 3MS is not strictly comparable either with the MMSE or with other standard cognitive tasks.

In September 1986, before the publication of the 3MS, the MRC convened an Alzheimer's Disease Workshop[11] whose aim was to establish the minimum dataset that should be collected in research studies on dementia. This included demographic data, history of physical and psychiatric disorder, alcohol and drug use, onset and duration of any difficulties, a physical examination, and a cognitive assessment. For the cognitive assessment, the MRC report recommended using the standardised administration and scoring instructions for the community version of the MMSE[10]. In addition, the MRC report recommended including the following items: category fluency (animal naming), recalling a name and address, assessment of remote memory, assessment of recent memory, ideational praxis, abstract thinking (similarities), and recognition of objects from unusual views. The specific aim of the additional items was to broaden the coverage of the MMSE in relation to both content and level of difficulty. The content requirement was to meet the needs for diagnostic criteria for dementia by including measures of perception and executive function; the difficulty requirement was to meet the need to differentiate between scores at the high end of the ability range. Individuals whose premorbid cognitive ability was high, might continue to obtain high scores on the MMSE and thus be missed on the MMSE screening test, even though their ability had in fact declined. It was hoped that the EMSE would measure more readily at higher cognitive abilities, and thereby differentiate between individuals at the high end of the ability range, therefore there would be scope to detect decline from an initially high level of functioning.

The MMSE items and most of the additional cognitive items recommended by the MRC report were included at the screening stage of the multi-centre MRC Cognitive Function and Ageing Study[12] and the findings are reported here. The screening test also included a measure of prospective memory (remembering to carry out an action), which has been reported elsewhere[13].

The aim of this paper is to describe the population distribution of performance on the MMSE and an extended cognitive assessment (EMSE) in a representative UK population.

## Methods

### Study design and population

The Medical Research Council Cognitive Function and Ageing Study (MRC CFAS) is a longitudinal population-based cohort study that involves six different study centres. The six centres were chosen because they represent the main national variation with regards to urban-rural differences, the north-south and east-west gradients, and variation in socio-economic levels and in known rates of chronic disease. Furthermore, all centres had existing researchers who were experienced in conducting population-based studies of the elderly. Urban sites included Liverpool, Newcastle, Nottingham, and Oxford, and rural sites included Cambridgeshire and Gwynedd, in North Wales. Liverpool was not included in this particular analysis because it was funded earlier than the other sites and had a different design without the same extended measurement of cognition. The full study design of the five identical centres is described in detail elsewhere and is explained briefly here[12].

Random samples of subjects over the age of 65 were selected from the Family Health Service Authority lists, giving an interviewed sample of approximately 2,500 people in each centre, stratified for equal numbers aged 65–74 years and 75 years and above. The study is longitudinal and this analysis focuses on information obtained at baseline – the prevalence (first) wave of the study. There were two phases at the prevalence wave. The first, screening, stage was used to establish level of cognitive performance and baseline risk factors on all individuals. A median of three months later, 20% of the subjects had a more detailed assessment interview to establish dementia diagnosis using the Geriatric Mental State (GMS) Automated Geriatric Examination Computer Assisted Taxonomy (AGECAT) diagnosis [14]. This group included the majority of individuals identified by the screening interview as potential cases of dementia, plus a random subset of the remaining population.

### Cognitive measures

The screening interview included the Mini-Mental State Examination (MMSE) in the version developed for field surveys[10]. Spelling 'WORLD' backwards as an alternative to serial sevens was omitted to enhance standardisation. This version forms part of the Cambridge Cognitive Examination within the CAMDEX interview [15] and detailed administration and scoring instructions have been published [16,17]. The screening interview also included a selection of additional questions recommended by the MRC Alzheimer's Disease Workshop[11] as described previously. Coverage of language skills was extended by adding two objects to be named to the original MMSE objects. Praxis was extended by adding writing to dictation (a name and address) to the MMSE item writing a sentence, which requires the ability to write and to generate a sentence [4]. The coverage of memory was extended with three additional items: (a) asking subjects to recall the four objects they had named earlier in the session; (b) asking subjects to recall the name and address they had written earlier in the session; (c) a set of five questions assessing semantic memory or general knowledge. Executive function was assessed using a category fluency task (naming animals in one minute) and two similarities items. Perception was assessed by showing three photographs of familiar objects taken from unusual angles. These photographs and several of the other additional items (animal naming, writing to dictation, recalling the name and address, and one of the similarities items) were taken from the CAMCOG, and details of administration and scoring can be found elsewhere [16,17]. The additional items combined with the MMSE comprise the Extended Mental Status Exam (EMSE). The MMSE and the set of additional items each have a maximum score of 30, bringing the maximum score for the EMSE to 60. Details of the items in the EMSE (with the MMSE embedded) are shown in the Table 1. The MMSE takes around 7 minutes to administer, the additional EMSE items about 3 minutes.

**Table 1:** The Extended Mental State Exam

**MMSE Question**	**Physical Item**	**Question**	**Points**
M		What is the name of this place/what is the full address?	1
M		What is the name of this city/town/village?	1
M		What day of the week is it today?	1
M		What is the date today (day)?	1
M		What is the date today (month)?	1
M		What is the date today (year)?	1
M		What is the season?	1
M		What is the country?	1
M		Name two main streets nearby (or near your home).	1
M		What floor of the building are we on?	1
M	P	What is this called? (pencil)	1
M	P	What is this called? (wristwatch)	1
	P	What are these called? (keys)	1
	P	What is this called? (envelope)	1
		Name as many different animals as you can think of in one minute.	5
M		Repeat 'No ifs, ands or buts'	1
		What are the four things you were asked to name a few minutes ago?	
	P	(pencil)	1
	P	(wristwatch)	1
	P	(keys)	1
	P	(envelope)	1
		Who is the Prime Minister?	1
		Who is the president of the United States of America?	1
		What are the colours of the Union Jack (prompt: our national flag)?	1
		Who was Neville Chamberlain?	1
		Who was Guy Burgess?	1
M		Repeat and remember these three words: apple, table, penny	3
M		Serial sevens	5
		What were the three words you were asked to repeat a little while ago?	
M		(apple)	1
M		(table)	1
M		(penny)	1
M	P	Read this and do what it says. (CLOSE YOUR EYES)	1
M	P	Copy this drawing. (a five sided figure)	1
M	P	Write a complete sentence.	1
		Follow these instructions:	
M	P	Take this piece of paper in your right hand	1
M	P	Fold the paper in half with both hands	1
M	P	Put the paper down on your lap	1
	P	Write down the following name address on this envelope: John Brown, 42 West Street, Bedford	2
		In what way are an apple and a banana alike?	2
		In what way are a boat and a car alike?	2
		What was the name and address you were asked to remember a short while ago?	
	P	(John)	1
	P	(Brown)	1
	P	(42)	1
	P	(West Street)	1
	P	(Bedford)	1
	P	What is the object in this picture? (shoe)	1
	P	What is the object in this picture? (spectacles)	1
	P	What is the object in this picture? (pipe)	1
		**Total**	**60**

At screen, subjects were assigned an organicity score using the organic symptoms component of the AGECAT computerised algorithm [14]. The AGECAT at screen uses nine questions to obtain a level of organic symptoms. This algorithm is based mainly on interviewer ratings and the only items common to the MMSE and AGECAT are orientation items (place name and address, and current date – day, month and year). The only further item in common between AGECAT and the EMSE is naming the current UK Prime Minister. The organicity score ranges from O0 to O5, with O3 indicating mild organic symptoms, and O4 and O5 indicating probable dementia diagnosis.

Twenty percent of the screened sample went on to the diagnostic assessment interview. This included the majority of those who had a screen AGECAT organicity score of O3 and above. Individuals with a score of O3 at screen could have had dementia, but they are a mixed group with mild organic symptoms that could relate to dementia, mild cognitive impairment or depression. Of this group, those who went on to receive a diagnosis of dementia at the assessment interview were regarded as demented for this analysis. All other individuals who scored O3 and the interviewer reported moderate to severe memory impairment were excluded from the analysis for non-dementia norms.

### Analysing performance on MMSE and EMSE

When describing performance on the MMSE and the EMSE, age was grouped into five-year bands (65–69, 70–74, 75–79, 80–84, 85 and above). Education was grouped into low level (9 years of schooling or less) and high level (greater than 9 years of full-time education). Those who had missing data about their educational attainment (n = 337) were placed in the low group.

### Normative tables

To derive normative data on the MMSE and the EMSE, individuals classified as demented at screen or at assessment were excluded (n = 627). Tables by age group and sex, as well as by age, sex, and education are presented.

### Missing data

Items that may have been missed due to sensory or motor impairment, called physical items, were recoded to 0 (i.e. treated as an incorrect answer). Such items include those involving writing or drawing, or those involving visual object recognition (see table 1 for details of items classified as being physical). Furthermore, in the MRC additional items, subjects are asked to recall an address that they have previously been asked to write. If the subject was physically unable to write the address, the instructions were to repeat twice by the interviewer and later the subject would be asked to recall it. There were a large number of missing values for these items, it is likely that interviewers omitted this recall in these individuals because of physical limitations. Therefore, the recall of the written address was categorised as a physical item and missing values were recoded to 0.

In general, individuals with items completely missing on the MMSE or the EMSE were not given scores for these tests. Many people were missing only one or two items from the MMSE or from the whole EMSE. Those missing two or fewer questions had their missing values recoded to 0 and were included in the analysis to ensure maximum use of the available data.

A sensitivity analysis to this missing data assumption has been undertaken using a pro-rata missing value score for the physical items. Individuals with missing data on physical items had a score generated for the proportion correct for their total score removing the physical question items from both the numerator and denominator.

### Factors influencing missing scores

In order to have a clear picture of the sample on whom we have cognitive data, it is important to compare them with the sample from whom we were unable to obtain data. The interview was designed such that there was a small subset of questions deemed 'priority' questions to be answered by all individuals, if at all possible. These included the AGECAT organicity screen and the MMSE items. The interviewer could request 'priority mode' at any time or it was selected automatically if an individual was not orientated to time or place. Hence, there are missing data by design that need further investigation.

Several potential factors were investigated to describe the differences between those individuals who had a complete EMSE score, those who only had a complete MMSE score, and those who had neither test complete. These factors included the demographic variables, gender, age, education and social class (as defined either by the respondent's current or last occupation, or for some women, by their husband's current or last occupation). Other factors included dementia status, whether the subject appeared to be muddled, and whether the interview went into priority mode or had to be abandoned. Physical health was also analysed in relation to missing data, and included ADL impairment using the Townsend disability scale [18]. Interviewer-reported language problems or speech impairment, and an interviewer and self-reported evaluation of hearing impairment, visual impairment, and whether or not the subject was chairbound or bedfast were also included. Self-reported health problems including heart attack, transient ischaemic event, stroke, diabetes, Parkinson's disease, angina as measured by the Rose angina questionnaire[19], smoking status, and global self-reported health (excellent/good/fair/poor) were analysed.

### Statistical Methods

Scores on the MMSE, and to a lesser degree the EMSE, do not follow a normal distribution. Hence, medians and other percentiles have been provided. For completeness, a logarithmic transformation (log (31-MMSE) or log(61- EMSE)) has been calculated and the estimate and reference ranges have been back-transformed to the original scale. Version 6.2 of the CFAS data has been used in this analysis. The analysis has been undertaken using STATAversion 8 [20].

## Results

The total number of individuals screened was 13,004, representing a response rate of 80%. Table 2 shows the total number of subjects screened, and those with the MMSE complete and the EMSE complete by age and sex (12,680 (98%) had complete MMSE data and 12,394 (95%) had complete data on both MMSE and EMSE). Fewer subjects completed the entire EMSE compared to the MMSE because if a subject was unable to complete other sections of the interview, they entered priority mode where only a subset of questions were asked and most of the MRC additional items were skipped.

**Table 2:** Total number of people screened, MMSE complete, and EMSE complete, by sex, age, and dementia status

	**Men**		**Women**		**Total**
**Age (years)**	**65–74**	**75+**	**65–74**	**75+**	
**Total screened**	2828	2329	3506	4341	13004
**Non demented**	2750 (97%)	2107 (90%)	3432 (98%)	3731 (87%)	12060 (93%)
**Demented**	78 (3%)	222 (10%)	74 (2%)	570 (13%)	944 (7%)

**MMSE score^†^**	2807	2272	3478	4123	12680
**Non-demented**	2735 (97%)	2076 (91%)	3410 (98%)	3664 (89%)	11885 (94%)
**Demented**	72 (3%)	196 (9%)	68 (2%)	459 (11%)	795 (6%)

**EMSE score^‡^**	2788	2216	3455	3935	12394
**Non-demented**	2729 (98%)	2062 (93%)	3400 (98%)	3632 (92%)	11823 (95%)
**Demented**	59 (2%)	154 (7%)	55 (2%)	303 (8%)	571 (5%)

^† ^Total number of respondents with complete MMSE score; ^‡ ^Total number of respondents with complete EMSE score

**Figure 1:**
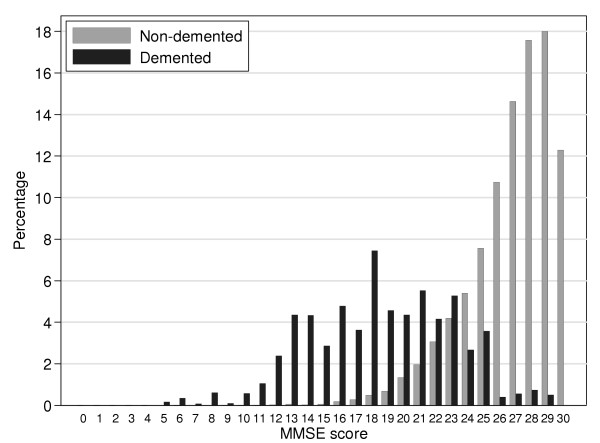
MMSE scores in the demented and the non-demented

**Figure 2:**
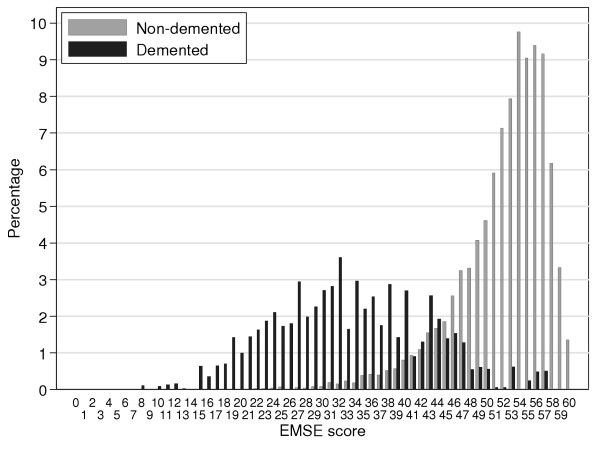
EMSE scores in the demented and non-demented

**Figure 3:**
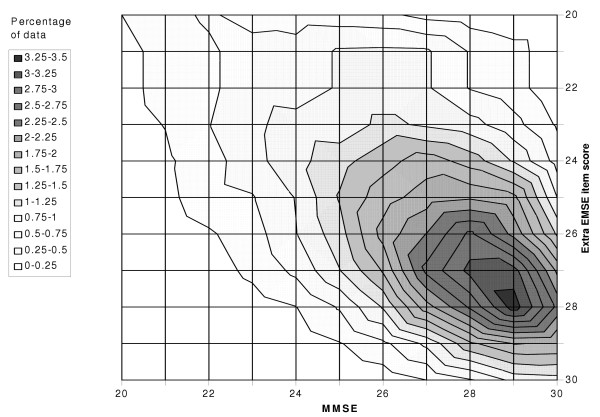
Joint relationship between top entries on the MMSE and extra items of the EMSE scale

For the sample as a whole, the median MMSE score is 27 (interquartile range 24 to 28) and the median EMSE score is 52 (interquartile range 47 to 55). The distributions of the two scores are presented in Figures 1 and 2, separately for the non-demented and demented groups. It can be seen that while the MMSE distribution is extremely skewed and truncated (Shapiro-Wilk W z = 18.5, skewness = -2.1), the skew is less marked for the EMSE (Shapiro-Wilk W z = 18.0, skewness = -1.6). The percentage of the sample who obtained maximum or near maximum scores (29 or 30) on the MMSE was 23.7% and the percentage who obtained maximum or near maximum scores (29 or 30) on the extra EMSE items was just 8.9%. Hence the EMSE shows better differentiation between scores at the high end of the scales. The Figures also show a relatively flat bell-shaped distribution of MMSE and EMSE scores for the group with dementia. The degree of overlap between the scores of demented and non-demented groups was similar in both MMSE and EMSE. A further illustration of the improved discrimination achieved by the EMSE at the high end of the distribution is depicted in Figure 3. This figure depicts the joint relationship between the high end of the MMSE scores (20–30) and the high end of the extra scores for the EMSE items (20–30). The contours show the increasing percentage of the study by score on MMSE and extra EMSE items. Whilst there is the expected relationship between ability on both parts of the EMSE scale the highest peak is centred just under 29 on the MMSE scale and 28 on the extra items. This shows the extent to which the EMSE successfully extends the scoring range for individuals who had already scored a maximum on MMSE.

**Figure 4:**
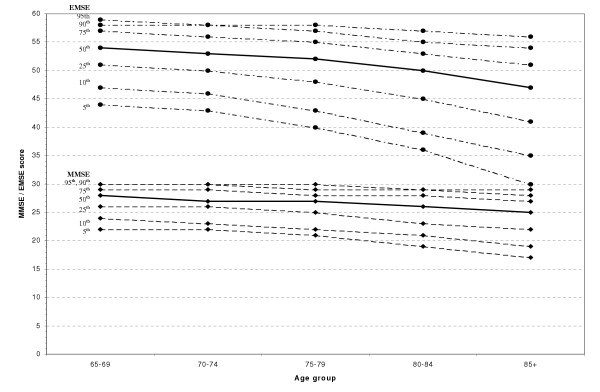
Percentiles for the MMSE and EMSE by age

Logarithmic means, standard deviations, medians, and various percentiles for both the MMSE and the EMSE in non-demented individuals are reported in Tables 2, 3, 4, 5, 6 and depicted graphically in figure 4. A logarithmic transformation [log(maximum score+1-score)] has been used to normalise the distributions of both measures, as they are both skewed. Both the MMSE and the EMSE have ceiling effects. The means and standard deviations were calculated on the log scale and the results were then back-transformed to the original scale for reporting purposes.

**Table 3:** Normative values for MMSE total score by age-group and sex (group without dementia)

	**Age-group (years)**				
	**65–69**	**70–74**	**75–79**	**80–84**	**85+**
**Men (total)**	1407	1328	1080	700	296
**Median**	28	28	27	26	25
**5^th^, 10^th ^pcntle**	23,25	22,24	21,23	21,23	18,20
**25^th^, 75^th ^pcntle**	27,29	26,29	25,29	24,28	23,28
**90^th^, 95^th ^pcntle**	30,30	30,30	29,30	29,29	28,29
Mean*	28.0	27.8	27.2	26.6	25.7
+/- 1 SD*	25–29	25–29	23–29	23–29	21–28
+/- 2 SDs*	20–30	18–30	16–30	16–30	11–30

**Women (total)**	1703	1707	1646	1257	761
**Median**	28	27	27	25	24
**5^th^, 10^th ^pcntle**	22,23	21,23	21,22	19,20	17,19
**25^th^, 75^th ^pcntle**	26,29	25,29	24,28	23,27	21,27
**90^th^, 95^th ^pcntle**	30,30	30,30	29,30	29,29	28,29
Mean*	27.9	27.5	26.8	25.7	24.7
+/- 1 SD*	24–30	24–29	23–29	21–28	19–28
+/- 2 SDs*	17–30	16–30	14–30	11–29	9–29

* Back-transformed; pcntle Percentile of the distribution; SD Standard deviation

**Table 6:** Normative values for EMSE total score by age-group and sex, and educational level (low: ≤ 9 years, high: > 9 years)

**Age (years):**	**65–69**		**70–74**		**75–79**		**80–84**		**85+**	
**Educational level:**	**L**	H	**L**	**H**	**L**	**H**	**L**	**H**	**L**	**H**

**Men**	913	491	812	513	693	379	425	277	199	94
**Median**	54	56	53	55	52	54	50	52	47	51
**5^th^, 10^th†^**	44,47	48,51	42,46	46,49	40,42	44,48	38,41	42,44	31,34	32,38
**25^th^, 75^th†^**	51,56	54,57	49,56	53,57	48,54	51,56	45,53	49,55	40,51	47,54
**90^th^, 95^th†^**	58,58	59,59	57,58	58,59	57,58	58,59	55,57	57,58	54,55	56,57
Mean*	54.2	55.8	53.3	55.4	51.8	54.5	50.2	52.5	47.2	51.0
+/- 1 SD*	48–57	51–58	46–57	50–58	44–56	48–58	42–55	46–56	36–53	43–55
+/- 2 SDs*	36–59	42–60	34–59	38–60	31–58	36–59	29–57	33–58	18–57	30–58

**Women**	1018	683	1011	688	1015	627	811	433	480	266
**Median**	53	55	52	55	51	53	47	51	45	49
**5^th^, 10^th†^**	42,45	46,49	41,44	46,48	38,42	42,46	33,37	39,42	29,33	35,38
**25^th^, 75^th†^**	50,56	53,57	48,55	52,57	46,54	50,55	43,51	47,54	40,50	43,53
**90^th^, 95^th†^**	58,58	59,59	57,58	58,59	56,57	57,58	54,55	56,57	53,54	56,57
Mean*	53.6	55.6	52.4	54.8	50.9	53.4	47.9	51.6	45.6	49.6
+/- 1 SD*	47–57	50–58	45–56	48–58	43–55	47–57	39–53	44–56	35–52	39–55
+/- 2 SDs*	33–59	39–60	31–59	35–59	29–58	34–59	23–57	30–58	17–56	20–58

^† ^Percentiles of the distribution; SD Standard deviation; * Back-transformed

Tables 3 and 4 present normative values for the MMSE and EMSE respectively, by age group and sex. All normative values are for individuals without dementia. There is a marked effect of age, with older subjects performing more poorly on both tests. The effect is particularly evident for the lowest percentiles (5^th^,10^th^) of the distribution, and for two standard deviations below the mean of the distributions. There is also a modest effect of gender, with women obtaining lower scores, which is particularly marked in the oldest age groups.

**Table 4:** Normative values for EMSE total score by age-group and sex (group without dementia)

	**Age-group (years)**				
	**65–69**	**70–74**	**75–79**	**80–84**	**85+**
Men (total)	1404	1325	1072	697	293
**Median**	54	54	53	51	48
**5^th^, 10^th ^pcntle**	44,48	43,46	41,44	38,42	31,35
**25^th^, 75^th ^pcntle**	52,57	50,56	49,55	46,54	42,52
**90^th^, 95^th ^pcntle**	58,59	58,59	57,58	56,57	55,56
Mean*	54.7	54.0	52.4	51.1	48.5
+/- 1 SD*	49–58	47–57	45–57	43–55	38–54
+/- 2 SDs*	37–59	34–59	31–59	30–58	20–57

**Women (total)**	1701	1699	1642	1244	746
**Median**	54	53	52	48	46
**5^th^, 10^th ^pcntle**	43,46	42,45	40,43	34,38	30,35
**25^th^, 75^th ^pcntle**	51,57	49,56	47,55	44,53	41,51
**90^th^, 95^th ^pcntle**	58,59	57,58	56,58	55,56	54,55
Mean*	54.4	53.3	51.8	49.2	47.0
+/- 1 SD*	48–58	46–57	44–56	40–54	36–53
+/- 2 SDs*	34–59	32–59	30–58	23–57	16–57

* Back-transformed; pcntle Percentile of the distribution; SD Standard deviation

The effect of education on cognitive performance can be seen in Tables 5 and 6 where scores are broken down by age group, sex and level of education. Level of education has a marked effect on both MMSE and EMSE scores for all age groups and for both sexes. Therefore, when education level is known, users of these tables are advised to consult Tables 5 and 6, as they provide a better estimate of the individual's expected level of cognitive ability.

**Table 5:** Normative values for MMSE total score by age-group and sex, and educational level (low: ≤9 years, high: >9 years)

**Age (years):**	**65–69**		**70–74**		**75–79**		**80–84**		**85+**	
**Educational level:**	**L**	H	**L**	**H**	**L**	**H**	**L**	**H**	**L**	**H**

**Men**	916	491	815	513	698	382	427	273	200	96
**Median**	28	29	27	28	27	28	26	27	25	27
**5^th^, 10^th†^**	22,24	24,26	22,23	23,25	21,22	23,24	20,22	22,24	18,20	19,21
**25^th^, 75^th†^**	26,29	27,29	26,29	27,29	25,28	26,29	24,28	25,28	22,27	25,28
**90^th^, 95^th†^**	30,30	30,30	30,30	30,30	29,30	30,30	29,29	29,30	28,29	29,29
Mean*	27.8	28.6	27.5	28.3	26.9	27.9	26.2	27.2	25.1	26.7
+/- 1 SD*	25–29	26–30	24–29	26–30	23–29	25–29	22–28	24–29	20–28	23–29
+/- 2 SDs*	19–30	21–30	18–30	20–30	15–30	20–30	16–29	18–30	11–29	14–30

**Women**	1019	684	1016	693	1018	628	815	442	491	270
**Median**	27	28	27	28	26	27	25	27	24	25
**5^th^, 10^th†^**	22,23	23,25	21,22	23,24	20,22	22,23	18,20	20,22	16,18	18,19
**25^th^, 75^th†^**	25,29	27,29	25,28	26,29	24,28	25,29	22,27	24,28	21,26	23,27
**90^th^, 95^th†^**	30,30	30,30	29,30	30,30	29,30	29,30	28,29	29,30	28,29	29,29
Mean*	27.6	28.4	27.1	28.0	26.5	27.5	25.2	26.7	24.2	25.7
+/- 1 SD*	24–29	26–30	23–29	25–30	22–29	24–29	20–28	23–29	19–27	21–28
+/- 2 SDs*	16–30	20–30	15–30	19–30	14–30	17–30	11–29	14–30	9–29	11–30

^† ^Percentiles of the distribution; SD Standard deviation; * Back-transformed

Using selected cut-points from these normative tables, we examined how well these values were able to differentiate between demented and non-demented groups. These data are presented in Tables 6, 7, 8 and 9. Table 8 examines absolute cut-points, without taking account of socio-demographic characteristics. The next table presents the results adjusted for age and sex, which is useful for cases where the level of educational attainment is unknown. Table 8 presents the data adjusted for age, sex and education. From these tables, it can be seen that the EMSE is no better than the MMSE at distinguishing demented from non-demented individuals. Roughly the same percentage of demented subjects fall below the 5^th ^percentile, the 10^th ^percentile, 1 standard deviation of the mean, and 2 standard deviations of the mean for the MMSE and the EMSE. Furthermore, when comparing three tables, we find that adjusting for age and sex makes little difference to the percentage of demented subjects who fall below the given cut-points. Further adjusting for education seems to have no added benefit in this context. This suggests that the EMSE is primarily extending the description of higher functioning individuals rather than discriminating between the low functioning groups.

**Table 7:** Number and percentage of people below given cut-points on MMSE and EMSE by dementia status

	**MMSE Non-demented**	**MMSE Demented**	**EMSE Non-demented**	**EMSE Demented**
**Below 5^th ^pcntle **(MMSE cut-point = 21, EMSE cut-point = 41)	618 (3%)	622 (73%)	810 (5%)	474 (77%)
**Below 10^th ^pcntle **(MMSE cut-point = 23, EMSE cut-point = 45)	1443 (8%)	707 (84%)	1676 (10%)	524 (88%)
**Below -1 SD **(MMSE cut-point = 23.1, EMSE cut-point = 44.5)	2075 (13%)	739 (90%)	1676 (10%)	524 (88%)
**Below -2 SD **(MMSE cut-point = 12.9, EMSE cut-point-24.5)	5 (0%)	229 (24%)	36 (0%)	125 (19%)

pcntle Percentile of the distribution; SD Standard deviation of the mean

**Table 9:** Percentage of people below given cut-points on MMSE and EMSE by dementia status, adjusted for age, sex, and education

	**MMSE Non-demented**	**MMSE Demented**	**EMSE Non-demented**	**EMSE Demented**
**5^th ^pcntle**	4	66	5	68
**10^th ^pcntle**	8	78	9	80
**-1 SD of the mean**	16	87	12	87
**-2 SD of the mean**	1	39	1	33

pcntle Percentile of the distribution; SD Standard Deviation

**Table 8:** Percentage of people below given cut-points on MMSE and EMSE by dementia status, adjusted for age and sex

	**MMSE Non-demented**	**MMSE Demented**	**EMSE Non-demented**	**EMSE Demented**
**5^th ^pcntle**	4	68	4	66
**10^th ^pcntle**	8	80	9	83
**-1 SD of the mean**	14	87	12	88
**-2 SD of the mean**	1	38	1	31

pcntle Percentile of the distribution; SD Standard Deviation

### Impact of missing data

Excluding the missing physical items responses from both the numerator and denominator caused little change in the results. The median and 5^th ^percentile MMSE scores were at most one point higher. The EMSE median scores were not affected, however the 5^th ^percentile was at most 2 points higher.

A comparison of the characteristics of those with complete MMSE scores, those with only complete EMSE scores and those with neither score complete has been undertaken. Those with no complete data or with only the MMSE complete were more likely to be older, female, in a manual occupation, and have a low level of education. They were also far more likely to have been classified as demented at screen. The vast majority of those who only had the MMSE complete (95%) went into priority mode during the screen interview. In general, those who completed both tests had fewer health problems than the other two groups.

## Discussion

This paper presents the full normative values for the MMSE in a UK population sample aged 65 years and over, together with normative values for an extended cognitive assessment (EMSE), with a more complete coverage of cognitive domains than the MMSE and a wider difficulty range. The normative values have been calculated for the whole population sample, excluding those with probable or diagnosed dementia. The MMSE norms adds to the existing literature, in both English and other languages, providing norms based on the largest study to date for this age group [21-24].

This paper also presents normative values for a new scale – the Extended Mental State Exam (EMSE) which combines the MMSE with additional items recommended by a MRC Alzheimer's Disease Workshop [11]. Results show that the EMSE is more normally distributed than the MMSE and avoids the ceiling effect known to impair the usefulness of the MMSE in measuring population levels of cognitive ability. Compared with the MMSE, where almost a quarter of the normative sample achieved the two highest scores (29 or 30), only 9% of the sample achieved a similar level on the extra items in the EMSE.

We have examined how well selected MMSE and EMSE values differentiate between individuals with and without dementia. Both perform moderately well for this purpose. Like the EMSE, the Modified Mini-Mental State (3MS) Examination [4,25] extends the coverage of the MMSE and produces a much wider range of scores. However, the 3MS does not cover one of the domains of cognitive function required for a diagnosis of dementia, i.e. perception, and the additional items are geared more towards extending the low end of the ability range than the high end. Although the 3MS incorporates all the MMSE items, some have been modified, which makes it difficult to compare them with standard MMSE scores (see Introduction). In contrast, the EMSE incorporates the standardised field survey version of the MMSE [10] and the additional items are also presented in a standard way, thus enhancing the comparability between the EMSE and other measures. However unlike the EMSE the additional questions in the 3MS have been shown to assist in differentiating between individuals with and without dementia[9].

The values of the population norms were affected by level of education as in other studies [21,22,26] however a somewhat unexpected finding was that using age-sex and education cutpoints did not improve the discrimination between the normal and non-demented groups, as found previously using the 3MS [27], but in contrast to other studies that have used MMSE [28,29].

Other researchers have investigated the use of the MMSE in individuals with physical impairments and suggested improvements [30], but in this large sample we did not find that adjusting for missing had much impact on the distributions for either the MMSE or EMSE due to the coding of the physical items. This effect has been seen with MMSE in other studies[31].

The results show that the EMSE is comparable to the MMSE in its ability to differentiate between individuals with and without dementia. However, as described above, the EMSE is superior at providing data for individuals at the high end of the performance range, in a similar way to other tests (e.g. TICS-M and Hopkins Verbal Learning Test [32,33]).

The choice of a screening test for dementia depends on the type of population to be screened, and the aim of the screening procedure. If the population to be screened can be expected to perform poorly on a cognitive function measure (e.g. hospital patients), then we believe that the EMSE has few advantages over the shorter MMSE. This is also true if the aim of screening is to pick up definite cases of dementia. However if the purpose of the cognitive test is to examine whether individuals have early cognitive changes or mild cognitive impairment (MCI) or when the population to be measured includes many high performing individuals (e.g. population surveys), then the EMSE applied longitudinally has distinct advantages over the MMSE, and the additional 3 minutes of administration time may be regarded as worthwhile.

The data reported here are from the first cross-sectional wave of the MRC Cognitive Function & Ageing Study [12]. The EMSE has also been administered at later waves of the study, and later papers will examine longitudinal aspects of the EMSE and its ability to detect new cases of dementia.

## Conclusion

Population-derived norms are valuable for comparing an individual's score to the score that would be expected among the general population, given the individual's specific demographic characteristics.

## Role of funding source

The funding bodies have had no influence on the paper or decision to publish.

## Conflict of interest

The author(s) declare that they have no competating interests.

## Contributions

FH oversaw the clinical context of the paper, SC undertook the initial statistical analysis and co-wrote the paper, FM oversaw the analysis, undertook the final analysis and co-wrote the paper. MRC CFAS investigators undertook the fieldwork and oversaw the scientific integrity of the study and commented on the paper. All authors have seen and approved the final draft of the paper.

## Pre-publication history

The pre-publication history for this paper can be accessed here:


